# Use of susceptibility scoring in conjunction with the genotypic transmission disequilibrium test

**DOI:** 10.1186/1471-2156-6-S1-S137

**Published:** 2005-12-30

**Authors:** Kelly S Benke, Gary A Chase, Daniele M Fallin

**Affiliations:** 1 Department of Epidemiology, Johns Hopkins Bloomberg School of Public Health, Baltimore, MD, USA; 2 Department of Health Evaluation Sciences, Penn State College of Medicine, Hershey, PA, USA

## Abstract

We explored the utility of selecting a genetically predisposed subgroup to increase the finding of a genetic signal in the Genetic Analysis Workshop 14 Collaborative Study on the Genetics of Alcoholism dataset. A subgroup of affected probands with low environmental risk exposures was defined using a susceptibility score calculated from an environmental risk model. Thirty-nine probands with highly positive scores were selected, along with their parents, for use in a genotypic transmission disequilibrium test (TDT) test. We compared the results of the genotypic TDT in this subgroup to the TDT results using all probands and their parents. For some markers, the susceptibility scoring approach resulted in smaller *p*-values, while for other markers, evidence for a genetic signal weakened. Further explorations into genetic and environmental population characteristics that benefit from this approach are warranted.

## Background

The most challenging and prevalent traits in genetic epidemiology today are complex phenotypes. Studies of such phenotypes are likely limited by genetic heterogeneity and high phenocopy rates, reducing power to detect linkage and association. Techniques to identify homogenous subgroups that harbor a genetic predisposition should involve the consideration of environmental influences. Accounting for non-genetic influences is crucial for the successful modeling of complex traits [1].

Family-based association tests (FBAT) have been used to detect association and linkage, or association in the presence of linkage. The original transmission disequilibrium test (TDT) considered affected children only [2]. A modified FBAT providing a unifying approach that builds on the TDT method allows, among other things, the consideration of nuclear families, unaffected family members, and parents with missing genotype information [3]. This modified FBAT has also been extended to include environmental covariates through the use of a covariate-adjusted phenotype, T [4], which is simply the subtraction of a continuous or dichotomous covariate from a dichotomous indicator of affection status.

Employing the modified FBAT method, Poisson et al. [5] developed a unique covariate-adjusted phenotype by conceptually defining and implementing a susceptibility score. The susceptibility residual is derived from a logistic regression model of affection status as a function of only known environmental predictors. This susceptibility residual is then used as the phenotype "T" in the FBAT procedure, as it is equal to the subtraction of the predicted value in the logistic model from affection status.

From this susceptibility score modeling, individuals with highly positive residual values represent those who are affected even though this would not have been expected on the basis of their environmental profile, while those with highly negative residuals represent those who are unaffected, even though they would have been predicted to be affected given their environment. Individuals with highly positive residual values, who show affection despite low environmental risk, may therefore harbor a genetic predisposition for the outcome. Use of these individuals for genetic analyses may then yield a stronger magnitude of effect among this subgroup.

When using these residuals as phenotypes "T" for FBAT, those with a residual close to zero do not contribute substantially to the test statistic. These individuals represent those with an environmental profile that predicts the outcome accurately, suggesting that genetic information would not provide additional information to an association test. Thus, it is unclear whether environmental information can increase power to detect linkage or association in this setting by reducing noise, or whether environmental information can reduce power by effectively lowering the contribution of persons with high genetic and environmental risk. In fact, Poisson et al. found that the use of the adjusted FBAT method involving the susceptibility residual oftentimes reduces the ability to detect a genetic signal.

To further explore the trade-off between an increased magnitude of effect and loss of power, we have used the susceptibility scoring approach to define a highly predisposed genetic subgroup, and compared results of a genotypic TDT on the subgroup to the entire group. Our strategy differs from the approach taken by Poisson et al. in that we used susceptibility scoring to reduce our analyses to a stratified subgroup, whereas the Poisson et al. paper utilized the entire sample, and compared adjustment of susceptibility score to non-adjustment.

## Methods

We used cleaned Illumina data from the Genetic Analysis Workshop 14 (GAW14) Collaborative Study on the Genetics of Alcoholism dataset. This dataset provided 137 families with a proband who was diagnosed with alcoholism according to DSM-IIIR + Feighner criteria and had genetic data available. Of these 137 families, 73 probands also had two parents with genetic data. The program FBAT was run for all family members relating to these 73 probands for all single-nucleotide polymorphisms (SNPs) provided for chromosomes 4 (275 SNPs), 15 (166 SNPs), and 16 (162 SNPs). These chromosomes were chosen due to previous suggestive linkage findings [6-8]. The 73 families range from 5 to 30 members and span 2–4 generations. FBAT tests the null hypothesis that there is no linkage or association, and thus treats nuclear families within the same pedigree independently. Markers that yielded *p*-values below 0.05 were then selected for the genotypic TDT test. Thus, the FBAT procedure was used as an initial screen to increase the opportunity of observing a SNP in linkage disequilibrium with a risk variant in subsequent genotypic TDT analyses.

The genotypic TDT focuses on the case, or child, genotype. Three pseudo-controls that represent the other possible genotypes that could have occurred conditional on the parental genotypes are matched to each case genotype. Conditional logistic regression was then performed on the 3:1 matched dataset in SAS v8.2 using the PHREG procedure. Beta coefficients can be interpreted as the change in log odds for the outcome associated with the possession of one copy of the 2 allele compared to the 1 allele and reflect an additive model. We conducted a genotypic TDT test for the 73 probands with parental genetic information, and on a subset of 39 probands that yielded a susceptibility score above 0.30.

To determine the susceptibility score for each proband, we selected all full siblings and modeled alcoholism as a function of age at interview, sex, ethnicity, and EEG phenotypes. To account for the correlation among siblings within families for alcoholism, we used generalized estimating equations specifying an exchangeable correlation structure. The difference between observed and expected values for each proband from this model is then the susceptibility residual. Susceptibility modeling was conducted in SAS v8.2 using the GENMOD procedure.

## Results

Table 1 shows the comparison of *p*-values between genotypic TDT analysis run with all 73 potentially informative trios versus the same test using a maximum eligible dataset of 39 trios based on a cutoff of 0.3 for genetic susceptibility. The actual number of trios utilized for each SNP is shown in Table 1. Red numbers are used to indicate larger *p*-values attributable to this restriction, and blue numbers specify those cases in which susceptibility scoring resulted in smaller *p*-values. We colored in only those rows where at least one *p*-value was less than 0.05 and the difference in *p*-values between the two methods was more than 0.01. Color was determined by the relative magnitude of the attained significance level. We also created susceptibility scores using the DSM-IV criteria for diagnosing alcoholism. Only two additional trios were gained, and results did not change meaningfully. Failure to include a significant predictor in the susceptibility model also did not alter the patterns observed in Table 1.

## Discussion

Our results are consistent with previous findings of Poisson et al., in which a covariate-adjusted phenotype, the susceptibility residual, was used in the FBAT procedure. These results were compared with results using an unadjusted phenotype for several simulated combinations of environmental and genetic main effects. It was found that power was consistently reduced when using the susceptibility score, while type I error was unaffected. However, when compared in real data, the disadvantage of the susceptibility score adjusted phenotype versus the unadjusted phenotype was unclear, possibly due to the unknown strength of the genetic effect, or interaction between genes and environment.

From our analysis of these GAW14 data, the overall picture again appears mixed, replicating the findings from Poisson et al. in a new context. This procedure is slightly different from Poisson's method but reflects the same underlying conceptual approach. Unlike Poisson, we are restricted in our ability to make direct inferences about power and type I error. The ideal design in which to apply our method would be a set of simulations in which the true variant is known. Our approach in the applied GAW14 dataset, however, provides some intuition for the main conclusions of a simulation approach.

The reduction of our susceptibility scoring approach to 39 high-risk trios led to a very small sample size. It is clear that the FBAT screening procedure led to a subset of markers with a greater number of significant *p*-values than expected as shown in Table 1 for the 73 trios. Reduction of the sample to the 39 trios did not result in more significant values than would be expected under the null. Datasets with a larger initial number of trios would have been more desirable. This finding, however, supports our conclusion that in most scenarios, any gain from defining a highly predisposed genetic group is offset by the loss in sample size.

Failure to measure informative covariates might have weakened the strength of susceptibility scoring. In complex diseases including alcoholism, genes and environment may both be active in transmission of a trait from parents to offspring. It is therefore important to distinguish correlation of genetic and environmental precursors of a complex trait within individuals versus gene × environment interaction effects on risk. For example, the risk factors might act independently on the trait but occur in non-random combinations. This problem could affect the relevance and impact of susceptibility scoring as well as other covariate adjustment procedures.

The nature of any gene × environment interactions would be important determinants of the relevance of the susceptibility scoring method. To understand more fully the utility of our susceptibility score approach, simulations of varying underlying gene × environment scenarios seem most informative. Gene × environment interactions of a synergistic type may tend to reduce the value of susceptibility scoring because of the probable discounting of highly informative transmissions. If those with high genetic susceptibility are rarely exposed to environmental risk, however, this method is likely to give favorable results by down-weighting potential phenocopies.

## Conclusion

Susceptibility scoring is a mixed blessing in that it confers benefit in cases in which the amount of information contributed by highly susceptible persons outweighs the sample loss from use of a cut-off point. Further study is needed to identify scenarios in which susceptibility scoring is most useful.

## Abbreviations

GAW14: Genetic Analysis Workshop 14

FBAT: Family-based association tests

SNP: Single-nucleotide polymorphism

TDT: Transmission disequilibrium test

## Authors' contributions

KSB carried out analyses and drafted the manuscript. GAC and MDF aided in the design of the analysis and provided intellectual input for the discussion. All authors read and approved the final manuscript.

## Figures and Tables

**Table 1:** Genotypic TDT results among the 73 trios and the 39 high susceptibility scoring trios

	No. of information families		Beta, *p*-value	
		
SNP (heterozygosity)	among 73 trios	among 39 high risk trios	73 trios	39 trios whose susceptibility residuals are above 0.30
Chromosome 4				
rs207338 (0.50)	57	29	0.29514, 0.1966	0.11173, 0.7092
rs1965907 (0.50)	54^a^	29	-0.58566, 0.0157	-0.64366, 0.0472
rs1040288 (0.50)	51	27	-0.36050, 0.1447	-0.23231, 0.4968
rs1478224 (0.48)	55	28	-0.70536, 0.0044	-0.54712, 0.1042
rs1472370 (0.50)	57	29	-0.37529, 0.1142	-0.58778, 0.0783
rs2178299 (0.44)	49	24	-0.36756, 0.1674	-0.59887, 0.1288
Chromosome 15				
rs965471 (0.47)	51^b^	28	-0.55324, 0.0318	-0.82098, 0.0233
rs1648308 (0.50)	53	28	0.74246, 0.0049	1.09121, 0.0045
rs1648312 (0.50)	53	28	-0.82091, 0.0019	-1.17132, 0.0023
rs2014638 (0.50)	62	33	0.22641, 0.3153	0, 1.0
rs1858359 (0.49)	57	27	0.29755, 0.2152	0.39855, 0.2627
rs725463 (0.50)	48	24	0.50450, 0.0665	0.83387, 0.0485
rs749468 (0.50)	52	29	0.28379, 0.2770	0.24181, 0.4905
rs872263 (0.20)	32	14	0.27193, 0.4125	-0.11765, 0.8087
rs2046071 (0.50)	56	30	0.51080, 0.0421	0.30718, 0.3628
rs1021393 (0.50)	58	29	0.06780, 0.7713	-0.05634, 0.8667
Chromosome 16				
rs8466 (0.31)	37	15	0.17588, 0.5816	0.54753, 0.3151
rs1019141 (0.50)	55	31	0.14948, 0.5223	0.20196, 0.5019
rs904821 (0.49)	50	23	0.38778, 0.1409	0.25025, 0.5113
rs991911 (0.48)	57	32	-0.33455, 0.1675	-0.33946, 0.2794
rs41383 (0.50)	52	28	-0.78702, 0.002	-0.55849, 0.1021
rs1541979 (0.47)	47	27	-0.34302, 0.1836	-0.25783, 0.4246
rs1074963 (0.47)	52	28	0.27269, 0.2585	0.31063, 0.3595
rs1037973 (0.47)	46	25	-0.31845, 0.2352	-0.43531, 0.2606
rs873857 (0.48)	47	24	0.11185, 0.6588	-0.35322, 0.3275

^a^Red text indicates susceptibility scoring results in larger *p*-values.

^b^Blue text indicates susceptibility scoring results in smaller *p*-values.
